# Responses of fitness center employees to cases of suspected eating disorders or excessive exercise

**DOI:** 10.1186/s40337-020-0284-9

**Published:** 2020-03-02

**Authors:** Flora Colledge, Robyn Cody, Uwe Pühse, Markus Gerber

**Affiliations:** Department of Sport, Exercise and Health, University of Basel, Birsstrasse 320b, 4052 Basel, Switzerland

**Keywords:** Eating disorder, Exercise, Fitness, Fitness center, Health, Unhealthy behaviours

## Abstract

**Background:**

While exercise and physical activity are important parts of a healthy life, there is evidence that some individuals exercise to a degree which may jeopardize their health. These individuals may in some cases be exercising to lose weight or compensate binge eating episodes as part of an eating disorder. Others may experience an addiction-like relationship with exercise. Fitness center employees are ideally placed to observe these forms of unhealthy behavior, and are responsible for ensuring that clients do not put themselves at undue risk; however, to date, no study has addressed both eating disorders and excessive exercise. Therefore, the aim of our study is to determine whether these employees report incidences of these issues, and if they believe they can differentiate between them.

**Methods:**

One-hundred-and-forty fitness centers in the German-speaking regions of Switzerland were contacted. Of these, 99 employees (60 men, 39 women, M_age_ = 33.33 years, SD = 12.02) responded to an online questionnaire. The questionnaire briefly described the two issues of interest (eating disorders and excessive exercise), and then invited respondents to complete a number of questions detailing whether they had experience with these issues, and how they dealt with them.

**Results:**

Approximately 75% of the employees had suspected a client of having an eating disorder or exercising excessively, and 65% of these respondents confronted the client at least once. Interestingly, respondents reported clearly that they felt able to differentiate between the two types of disorder. Older respondents were significantly more likely to have suspicions and act on them. However, less than half of the respondents were aware of guidelines addressing this issue, and the majority desired more information about how to identify and address both disorders.

**Conclusions:**

Swiss fitness center employees frequently encounter individuals who they suspect of exercising excessively, or suffering from an eating disorder. While they often confront these individuals, they would like more detailed information about how to manage this process. Given that both disorders can potentially lead to severe health consequences, a detailed description of symptoms, management techniques and resources should be a feature in all Swiss fitness center guidelines.

## Plain English summary

Although exercise and sport can be healthy, some people may do so much that it has unhealthy consequences. People with eating disorders or who feel addicted to exercise fall into this group. Often, these people will exercise in fitness centers. Fitness center employees might notice these people, and wonder if, and how, they should intervene when they are concerned about their health. This paper reports on how a group of fitness center employees in Switzerland react when they suspect clients might have an eating disorder or exercise too much. Most fitness center employees had experienced this, and many intervened, but they still felt that more guidelines about how to act would be helpful. The study participants felt that they could tell the difference between people exercising too much who did have an eating disorder, and those who didn’t, which is the first piece of evidence that fitness centre employees are confronted with both of these issues. More detailed guidelines to support fitness centre employees should address these two distinct issues.

## Background

Physical exercise is an important element of healthy human life. However, exercise can also have unhealthy consequences when engaged in excessively and without proper recovery [1]. For example, individuals with eating disorders may exercise in order to lose weight, or purge calories consumed. Individuals may also exercise excessively (beyond the requirements for general health or athletic adaptation) and find themselves unable to control or reduce the exercise volume [2].

It is well established that exercise is used as a weight control method by individuals with eating disorders, particularly anorexia nervosa and bulimia nervosa [3, 4]. Excessive exercise has not yet been classified as a behavioral addiction, compulsive disorder, or other form of psychiatric disorder [5]; however, the existing body of evidence suggests that there are individuals who exercise to a degree that causes them psychiatric distress, but they feel unable to stop (due to a sense of guilt at not completing planned sessions, or fear of severe withdrawal symptoms) [6]. It has been suggested that excessive exercise only occurs as a symptom of eating disorders [7]; however, numerous case studies indicate that there appear to be individuals who exercise to excess, but are not primarily, or perhaps at all, motivated by weight or body image [8–10]. Indeed, some researchers have posited a distinction between “primary” and “secondary” exercise addiction, with the primary form characterized by exercise itself as a rewarding experience to be pursued, while in the secondary form, another goal, such as weight loss or relief of anxiety is desired [11]. Due to the lack of clarity around the nature of excessive exercise, we treat this as a separate condition in this manuscript, but refrain from using terms such as “exercise addiction” or “exercise dependence”.

Certain eating disorders [12] and excessive exercise [13] involve a number of signs and behaviors that can be recognized by observers (although these are certainly not sufficient for a conclusive diagnosis). When these behaviors are carried out in a fitness center, it is therefore possible that the employees of that center are in a position to observe these signs [14]. Anorexia nervosa, particularly in an advanced stage, is likely to leave the sufferer at a very low body weight [15]; lanugo may also be visible [16]. Bulimia nervosa may be accompanied by the presence of Russel’s sign on the hand [17]; however, these latter two symptoms are unlikely to be recognized without specific training. Other forms of disordered eating, such as binge eating disorder or orthorexia, may involve no clear physical signs [18]. Indeed, signs such as overweight, in the case of binge eating disorder, may not be interpreted as symptomatic of an eating disorder at all. For this reason, although anorexia nervosa is not the most prevalent eating disorder [19], it may be the most widely recognized based on physical characteristics alone; so-called “weight bias”, the negative attitude towards overweight and obesity, and a corresponding tendency to overlook eating disorders which result in overweight, may also be relevant here [20].

Excessive exercise involves prolonged and regular bouts of physical exercise, a behavior which is potentially apparent to an observer, but which may be seen as healthy and laudable, or the preparation of a high-level athlete [21]. This can be distinguished from compulsive exercise, characterized by an extreme urge to exercise [3]; excessive exercisers may be motivated by this urge, but motivation is not inherent to the definition. There is evidence that individuals who exercise excessively are supported by their friends and family in this behavior [22]. There is also evidence that this behavior is carried out, at least to some extent, in fitness centers. Estimates of between 8% [23] and 43% [24] of “exercise dependence prevalence” have been reported, with Stapleton and colleagues [25] reporting at least a single occurrence in 43% of their male gym-using sample.

While studies for the prevalence of disordered eating amongst fitness center clients are scarce, it is estimated that between 20 and 50% of females with eating disorders engage in excessive exercise [3, 11]. There is also evidence that prevalence of eating disorders among such exercisers is higher than among competitive and organized sports participants [26]. Muller and colleagues reported that 10.9% of their sample of fitness center clients fulfilled the criteria for an eating disorder, as assessed by the Eating Disorders Examination-Questionnaire [23]. Stapleton and colleagues [25] reported at least one occurrence of binge eating or dietary restraint in 35 and 15%, respectively, of male fitness center clients. While exercise in any setting can be a means to lose weight, at least one study of 60 female aerobic exercisers has reported that exercise in the fitness center setting is associated with higher levels of self-objectification, which in turn was related to disordered eating habits [27]. Finally, Lejoyeux and colleagues [24] also reported that 57% of their sample of fitness center clients fulfilled Diagnostic and Statistical Manual of Mental Disorders (DSM) IV criteria for bulimia.

In summary, sufferers of both eating disorders and excessive exercise may display signs and behaviors of their disorders which could arouse suspicion in observers. It is likely that at least some sufferers of these disorders carry out some of their exercise in fitness centers. The majority of fitness centers employ staff and personal trainers who are responsible, among other duties, for ensuring that their equipment is used safely. It is likely that certain individuals suffering from exercise-related psychiatric disturbances and disorders will spend some time in fitness centers, and they may display symptoms that an attentive observer could pick up on. Fitness center employees are well placed to observe these symptoms.

In line with this notion, in a study of 143 Canadian fitness center employees, 62% reported having observed a client that they believed suffered from anorexia nervosa [28]. However, only just over 25% have been trained on how to deal with such a situation. Bratland-Sanda and Sundgot-Borgen [12], in their study of 837 Norwegian fitness instructors, reported that while 89% of instructors self-reported knowledge about eating disorders, only 29% were found to have adequate knowledge. While the authors do not speculate on the causes of this discrepancy, it may be a further example of the role of weight bias, with employees believing that physical appearance is a key indicator, rather than other signs and behaviors. Similarly, Manley, O’Brien and Samuels [29] reported that 32% of fitness instructors, compared with 88% of pediatricians, were accurately able to identify a case of anorexia nervosa presented in a case study. In their study of eating disorder characteristic recognition, Worsfold and Sheffield [30] reported that 80% of fitness instructors failed to recognize that a case study involved symptoms of an eating disorder. By contrast, only 40% psychologists failed to identify this. The authors also note that 36.7% of fitness instructors suggested that the behavior of the individual presented in the case study was desirable in achieving weight loss goals.

To date, no study has examined whether fitness center employees suspect that certain clients may be exercising excessively. As this behavior is still not an established psychiatric condition [5], it is also unclear whether fitness center employees are aware of it, or if they differentiate between excessive exercise and exercise related to an eating disorder. As the debate about the definition and categorization of excessive exercise habits is ongoing [31], fitness center employees represent an important source of information about the possible behaviors and indicators which may be observed in excessive exercisers. Crucially, they may also provide insight into whether these behaviors appear to differ from those shown by individuals who they suspect of eating disorders.

The aim of this study is therefore to determine whether fitness center employees in Switzerland believe they have observed clients who they suspect had an eating disorder, or were training excessively. While a small number of studies have examined employee responses to suspected eating disorders, none have to date addressed excessive exercise. Consequently, our study not only provides further insight into current approaches to two distinct conditions, but allows an understanding of how fitness professionals view the differences between the two, for instance by asking whether they have suspicions regarding the motivations of those they observe. Our study is the first to assess both disorders in a single questionnaire. By including excessive exercise, we hope to target the perspective of fitness professionals and gain valuable insight into a condition that is frequently only addressed from the psychiatric perspective.

This study does not aim to establish causal relationships between fitness center attendance and exercise-related disorders. We do not make statements about the degree of responsibility that fitness center employees have with regards to psychiatric conditions. This study is intended to be an exploration of the current behavior and concerns of fitness center employees.

## Methods

### Participants and procedures

The contact details of fitness centers in the German-speaking parts of Switzerland were identified via an online search. The terms “Fitness” OR “Fitnesscenter” AND “Schweiz” were used in the Google search engine. Based on the results, individual fitness centers were identified. Business listings and directories were also identified during this search, and these listings were scanned to ensure that the maximum number of fitness centers was identified. The email address and telephone number of all identified centers were collected, where possible. In many cases, no email address was available, but an online contact form in the homepage was provided. A cover letter was created, inviting the recipient to circulate the email internally, so that all interested fitness center employees could respond.

Two emails, separated by 2 weeks, were sent to all identified centers, either via the email address provided, or through the online contact form, in October and November 2018. A week after the second email, a telephone call was made to each center to request the circulation of the original email.

Email invitations were sent, and subsequent telephone calls made, to 140 fitness centers. In 18 cases, no one answered the telephone. In seven cases, the center stated that they did not wish to take part in the study. In all other cases, the center confirmed that they would forward the email to their employees. The local ethics committee, the Ethikkommission Nordwest- und Zentralschweiz, reviewed the study and granted an exemption from full ethical review, as only fully anonymized data would be collected (study request number Req-2018-00297).

### Instruments

A questionnaire was designed by the investigators. The questionnaire was created using the online software Umfrageonline (enuvo GmbH, Zurich, Switzerland). The first page informed participants about the anonymous nature of the survey, and explained that the questions concerned “unhealthy behavior” in fitness centers. A brief definition of eating disorders (“A psychiatric disorder characterized by disturbed eating patterns, which can be accompanied by an unrealistic body image”) and excessive exercise (“A condition characterized by a strict adherence to a program of exercise which goes beyond what is required for physical health, and may be psychologically damaging”) was provided.

Demographic data concerning age, gender, years working in the fitness industry, and educational level were gathered. Participants were also asked whether the center in which they currently worked was open to men and women, or restricted to one gender. Finally, we asked participants if they themselves had ever suffered from an eating disorder, or exercised excessively.

For the main body of the questionnaire, all questions contained separate response categories for eating disorder and excessive exercise. Participants were asked whether they had ever observed any individuals training in their gym who they suspected of suffering from either of these disorders. Participants who answered “yes” to one or both disorders were then asked a series of questions concerning the basis of their suspicions, how they reacted, and what effect their reaction had, if any. Participants who answered “no” regarding both disorders skipped the questions concerning suspicions, and were directed to the final section of the questionnaire, concerning guidelines on the management of suspected cases of these disorders. Participants who did have suspicions were also subsequently directed here.

The final section of the questionnaire asked participants to state whether they knew of any guidelines on how to manage suspected cases of these disorders, the source of these guidelines (e.g. organizational, national, international), whether they were satisfactory, and what information, if any, they would like to see included in future guidelines.

The questionnaire is based on instruments used in similar studies on this topic [12, 28]. It has not been validated or otherwise assessed. The results presented here must consequently be considered to be exploratory descriptive findings.

### Statistical analyses

All data analyses were carried out with SPSS Version 24 (IBM, Armonk, USA). Sample characteristics (Table 1), frequency of observed disorders and reasons for suspicion (Table 2), as well as desired guideline content (Table 3) are presented as absolute numbers (n) and percentages (%). We used χ2-tests, one-way ANOVAS and, when controlling for covariates, multiple logistical regression to examine whether (a) the observation of the two disorders, (b) the likelihood of confronting a client, and (c) awareness of existing guidelines were associated with the gender, age, education, self-report of suffering from either disorder and professional experience of the fitness center employees. *P*-values < 0.05 were considered as statistically significant differences.

**Table 1:** Demographic data of sample

*n* = 99		n (%)
Gender	Male	61 (61.6)
	Female	38 (38.4)
Age	Under 25 years	31 (30.7)
	25–49 years	54 (54.5)
	50 years or older	14 (14.8)
Education/Training	University degree	22 (22.2)
	Vocational training as a fitness instructor or employee	43 (43.4)
	Swiss professional certification system	28 (28.2)
	Other	6 (6.6)
Time in the fitness industry	Less than 5 years	41 (41.4)
	5 to 9 years	24 (24.2)
	10 years or more	34 (34.3)
Self-reported suffering from an eating disorder	Yes	8 (8.1)
	No	91 (91.9)
Self-reported suffering from excessive exercise	Yes	15 (15.2)
	No	84 (84.8)

**Table 2:** Reasons for suspicion and confrontation, separately for eating disorders and excessive exercise

Eating disorders			Excessive exercise		
*n* = 82		n (%)			n (%)
Reasons for suspiscion	Body shape	63 (76.83)	Reasons for suspiscion	Body shape	28 (33.15)
	Mood	25 (30.49)		Mood	26 (31.71)
	Clothing	8 (9.76)		Clothing	6 (7.32)
	Duration of training	21 (25.6)		Duration of training	54 (65.85)
	Frequency of training	32 (39.02)		Frequency of training	65 (79.27)
	Type of training	11 (13.41)		Type of training	22 (26.83)
	Physical signs	55 (67.07)		Physical signs	20 (24.39)
	Behavior	30 (36.59)		Behavior	30 (36.59)
*n* = 59		n (%) M			n (%) M
Types of confrontation	Asked how they were feeling	38 (64.40) M = 5.5	Types of confrontation	Asked how they were feeling	38 (64.40) M = 5.24
	Suggested they stop the session	24 (40.67) M = 1.8		Suggested they stop the session	26 (44.06) M = 1.65
	I showed concern for their health	43 (72.88) M = 4.32		I showed concern for their health	38 (64.40) M = 4.76
	I gave them health information	42 (71.18) M = 4.24		I gave them health information	39 (66.10) M = 5.10
	I suggested they take a few days off training	25 (42.47) M = 3.04		I suggested they take a few days off training	36 (61.01) M = 4.77
	I suggested they leave the gym for the day	23 (38.98) M = 0.86		I suggested they leave the gym for the day	24 (40.67) M = 1.58
	I forbid them to visit the gym for a few days	29 (49.15) M = 0.93		I forbid them to visit the gym for a few days	23 (38.98) M = 1
*n* = 13		n (%)			n (%)
Reasons for avoiding confrontation	I told my boss	5 (38.46)	Reasons for avoiding confrontation	I told my boss	2 (15.38)
	It’s not my responsibility	2 (15.38)		It’s not my responsibility	2 (15.38)
	I didn’t know what to say	5 (38.46)		I didn’t know what to say	5 (38.46)
	I was worried I would make things worse	3 (23.08)		I was worried I would make things worse	1 (7.69)
	Our guidelines forbid it	–		Our guidelines forbid it	–
	My boss/colleagues told me not to	–		My boss/colleagues told me not to	–

**Table 3:** Desired content of guidelines, separately for eating disorders and excessive exercise

Eating disorders			Excessive exercise		
*n* = 65		n (%)			n (%)
Desired guideline content	Information about signs and symptoms	45 (69.23)	Desired guideline content	Information about signs and symptoms	43 (66.15)
	Risks and consequences	36 (55.38)		Risks and consequences	35 (53.85)
	Concrete first steps for starting a dialogue	49 (75.38)		Concrete first steps for starting a dialogue	48 (73.85)
	How and when to give advice regarding stopping or modifying training	37 (56.92)		How and when to give advice regarding stopping or modifying training	37 (56.92)
	Contact information for helpful organizations	30 (46.15)		Contact information for helpful organizations	31 (47.69)
	Information about my responsibilities and duties	43 (66.15)		Information about my responsibilities and duties	40 (61.54)

## Results

### Sample characteristics

One hundred and one individuals responded to the survey. It is not possible to determine whether multiple individuals from a single center responded. Ninety-nine respondents completed all relevant questions, and their data are presented below. Demographic data are displayed in Table 1.

Ninety-six percent of fitness centers represented in this study are open to both men and women; 4 % are open to women only.

Seventy-seven percent of respondents reported having observed at least one client whom they suspected of having an eating disorder. Of these respondents, 13% reported that they had observed one client whom they suspected, 35% had suspicions about two to five clients, 22% had suspicions about six to 10 clients, 8% about 11 to 20 clients, and 12% about more than 20 clients. Seventy-three percent reported having observed a client whom they suspected of suffering from excessive exercise at least once. Similar to the rates reported for eating disorders, 5 % reported that they had observed only one client whom they suspected, 35% had suspicions about two to five clients, 21% about six to 10 clients, 12% about 11 to 20 clients, and 14% had suspicions about more than 20 clients. Older respondents were significantly more likely to report having suspected a client with an eating disorder (F(1,98) = 4.735, *p* = 0.032, η^2^ = 0.046) and exercise addiction (F(1,98) = 5.536, *p* = 0.021, η^2^ = 0.051). Longer time spent working in the fitness industry, however, did not show the same pattern. There was no association between gender, self-report of having either disorder, or educational background and the likelihood of suspecting a client of either disorder.

Sixty-three percent of respondents had confronted a client about a suspected eating disorder at least once. Sixty percent had confronted a client about suspected excessive exercise at least once. Older respondents who did suspect a disorder were also significantly more likely to confront an individual whom they suspected of having an eating disorder (F(1,82) = 6.576, *p* = 0.012, η^2^ = 0.074) or exercise addiction (F(1,82) = 4.850, *p* = 0.031, η^2^ = 0.058). In this case, longer time spent working in the industry also meant that respondents were significantly more likely to start confrontations in cases of suspected eating disorders (F(1,81) = 7.201, *p* = 0.009, η^2^ = 0.082) and exercise addiction (F(1,79) = 6.485, *p* = 0.013, η^2^ = 0.076). Gender, self-report of having either disorder, and educational background were not associated with the likelihood of confronting a client about either disorder. In total, 233 confrontations were reported with clients with suspected eating disorders. In 32% of cases, the client admitted that they had a problem. Regarding excessive exercise, 201 confrontations were reported. In 35% of cases, the client admitted that they had a problem. Reasons for confrontations (or avoiding them) are listed in detail in Table 2.

In total, 43% of respondents stated that they knew of guidelines which addressed clients with a possible eating disorder. Seventy-three percent reported that these guidelines were issued by the gym at which they were employed, 3 % knew only of other guidelines, and 24% knew of none. Among respondents who knew of guidelines, 63% reported that the guidelines gave them a lot of confidence, 32% reported gaining some confidence, and 5 % reported gaining a little confidence; no respondent reported that the guidelines gave them no confidence whatsoever. However, 84% of respondents reported that new or updated guidelines would be helpful in dealing with suspected eating disorders.

Forty-four percent of respondents stated that they knew of guidelines which addressed clients with possible excessive exercise. Whereas 73% reported that these guidelines were issued by the gym at which they were employed, 5 % knew only of other guidelines, and 21% knew of none. Among respondents who knew of guidelines, 69% reported that the guidelines gave them a lot of confidence, 29% reported gaining some confidence, and 5 % reported gaining a little confidence; no respondent reported that the guidelines gave them no confidence whatsoever. However, 86% of respondents reported that new or updated guidelines would be helpful in dealing with suspected cases of excessive exercise. Participants’ wishes regarding guideline content are shown in Table 3.

Older respondents were significantly more likely to know of guidelines on both eating disorders (F(1,76) = 5.164, *p* = 0.026, η^2^ = 0.064) and exercise addiction (F(1,74) = 8.077, *p* = 0.006, η^2^ = 0.098). Again, longer time in the fitness industry meant a significantly greater likelihood of knowing about guidelines concerning eating disorders (F(1,75) = 6.180, *p* = 0.015, η^2^ = 0.076) and exercise addiction (F(1,73) = 5.883, *p* = 0.018, η^2^ = 0.075). Gender and self-report of suffering from either disorder were not associated with knowledge of either type of guideline. However, interestingly, individuals who had completed a Bachelor or Master of Sports Science were less likely to be familiar with either type of guideline, compared to those who had completed vocational training, gone the federal professional certification scheme, or had some other form of training. Multinomial logistic regression, using age and time spent in the fitness industry as covariates, did not affect this relationship.

## Discussion

Our study is the first to address suspected cases of both eating disorders and excessive exercise in fitness centers, as evaluated by their employees. Overall, approximately 75% of respondents reported suspecting at least one client was affected by one of these disorders, with older respondents significantly more likely to suspect, intervene, and know of guidelines. This is a slightly higher rate than in similar studies set in Canada [28, 29] and Norway [12], in which 67, 62, 49% of respondents, respectively, suspected at least one client of an eating disorder (in our study the rate for eating disorders only was 77%). Our study, however, is the first to also assess suspicions of excessive exercise. Our results indicate that participants in this questionnaire felt that they were able to differentiate between these two conditions. Notably, participants stated that they suspected individuals of having an eating disorder primarily based on body shape and other physical signs, while suspicion of excessive exercise was primarily based on exercise duration and frequency. As it can be expected that many individuals who had no experience of these issues would simply ignore the questionnaire, this finding cannot be taken to indicate the prevalence of these disorders. Our study is therefore best understood as an indication of how fitness center employees who do have suspicions react to them. Below, we discuss the implications of our findings for the fitness industry.

A little under two thirds of respondents reported confronting individuals who they suspected of having an eating disorder or exercising excessively, a similar proportion to that reported in one study focusing on eating disorders, in which fitness center employees intervened 59% of the time [28]. While it is to be expected that a certain selection bias is at work, with employees who confront clients more likely to be interested in this questionnaire, it is interesting to note that almost 50% of respondents had confronted between two and ten clients. This may indicate that an individual who feels confident enough to confront a client once is likely to find the experience positive enough that he or she does not refrain from further confrontations. Again, only older age was significantly associated with greater likelihood of confrontation. Respondents used a variety of approaches, with the sole notable difference between the two disorders being the advice to take a few days off training. This advice was given in 42% of cases to individuals with a suspected eating disorder, and 61% of cases to individuals with suspected excessive exercise habits. This reinforces the distinction implied in employee assessments of signs of excessive exercise, namely, that volume plays a decisive role, while this appears to be seen as less of a problem in eating disorders. Interestingly while various attempts have been made to define and characterize excessive exercise, statements about specific training volume do not form part of these definitions. This is likely due to the fact that other factors more accurately express the “addictive” nature of exercise (for example, the rigidity with which a program is adhered to, difficulty in stopping despite social conflicts or health problems, etc.). By contrast, volume and frequency may only be troublesome or unhealthy in certain circumstances, for certain individuals – an unmarried professional athlete may train for twice the hours of an individual who exercises excessively, but the latter will be characterized as having a psychological problem because their training volume causes them to lie to their family, appear late for work, or continue exercising despite a high fever.

Similarly, there was one key discrepancy in reasons for avoiding confrontation; while just over 38% of respondents told their boss about a client with a suspected eating disorder, only just over 15% did this in the case of suspected excessive exercise. We can provide no evidence for the reason behind this discrepancy. Speculatively, it may be that as eating disorders are often accompanied by physical signs of poor health (and those which may not have been recognized by our respondents), a more pressing concern prompted employees to go to their superiors than in cases where clients were “simply” overdoing their training volume. While the physiological risks of anorexia and bulimia nervosa are well documented, the risks and effects of excessive exercise have not yet been systematically evaluated. Consequently, this uncertainty on the part of fitness center employees is in line with the current state of research regarding excessive exercise, and suggests that physiological, as well as psychological assessments may be warranted.

In the current study, less than half of respondents stated that they knew of guidelines which addressed either disorder. While two-thirds of these respondents found the guidelines they were aware of useful, the majority reported that new or updated guidelines would be helpful. In similar studies addressing eating disorders, the majority of respondents also wished for improved guidelines [28, 29] – a poll of fitness center employees assessing the information they deem valuable would seem to be an essential step for any national or international governing body. Interestingly, respondents with a university degree were significantly less likely to know of guidelines, a result not explained by age discrepancy. This is in contrast to the findings of Bratland-Sanda and Sundgot-Borgen, who found the higher educational levels linked to better identification of eating disorder symptoms [12]. We have not been able to identify any Swiss guidelines concerning eating disorders or excessive exercise amongst any exercising populations which are publicly available. Consequently, we are unable to comment on the content which our respondents are referring to. However, we have a limited indication of our respondents’ attitudes to this content, as we discuss below.

Regarding guideline content, there was little variation between the two disorders with regards to the information that respondents felt would be helpful. In both cases, the most commonly requested information concerned concrete examples of how to initiate dialogue in suspicious cases. A number of organizations in other countries issue guidelines which specifically offer this information. For example, Fitness Australia have collaborated with the Centre for Eating and Dieting Disorders to issue guidelines for managing gym members who may have eating or exercise disorders. This guideline document offers a detailed diagram of the steps involved in managing such cases, and lists strategies for engaging in conversation at each step [32]. The United States-based National Eating Disorder Association has released a toolkit for individuals who work with athletes, school sports programs, or in the fitness industry, which includes steps to take and examples of conversations [33]. However, the advice may be more applicable to coaches who work closely with an athlete, and a number of the steps involved, such as addressing eating behavior and social isolation, maybe be far outside of the purview of fitness center employees. The United Kingdom-based Institute of Sport and Recreation Management offers guidelines on managing clients who may have eating disorders [34]. While these guidelines do not provide detailed examples of conversational approaches, they also highlight the importance of creating a fitness environment which does not tacitly support unhealthy exercise habits. There are therefore a number of existing resources which could inform a new or amended guideline framework in Switzerland; we suggest that conversation examples and exercises which can be practiced by staff in advance would make a valuable addition to existing material.

## Conclusion

This study indicates that fitness center employees in Switzerland are sometimes confronted with individuals using their facilities who may be suffering from an eating disorder, or exercising to an unhealthy level. Respondents also felt able to differentiate between these two issues. While most confront the suspected person at least some of the time, more detailed guidelines, including examples for how to start conversations on this subject, are required.

## Data Availability

The datasets used and/or analysed during the current study are available from the corresponding author on reasonable request.

## Abbreviation

DSM: Diagnostic and Statistical Manual of Mental Disorders

## References

[1] Lichtenstein MB, Hinze CJ, Emborg B, Thomsen F, Hemmingsen SD (2017). Compulsive exercise: links, risks and challenges faced. Psychol Res Behav Manag.

[2] Meyer C, Taranis L, Goodwin H, Haycraft E (2011). Compulsive exercise and eating disorders. Europ Eat Disorder Rev.

[3] Dalle Grave R, Calugi S, Marchesini G (2008). Compulsive exercise to control shape or weight in eating disorders: prevalence, associated features, and treatment outcome. Compr Psychiatry.

[4] Davis C, Katzman DK, Kaptein S, Kirsh C, Brewer H, Kalmbach K (1997). The prevalence of high-level exercise in the eating disorders: etiological implications. Compr Psychiatry.

[5] Potenza MN (2014). Non-substance addictive behaviors in the context of DSM-5. Addict Behav.

[6] Szabo A, Griffiths MD, de La Vega MR, Mervó B, Demetrovics Z (2015). Methodological and conceptual limitations in exercise addiction research. Yale J Biol Med.

[7] Bamber DJ, Cockerill IM, Rodgers S, Carroll D (2003). Diagnostic criteria for exercise dependence in women. Br J Sports Med.

[8] Griffiths M (1997). Exercise addiction: a case study. Addiction Res.

[9] Spieker MR (1996). Exercise dependence in a pregnant runner. J Am Board Fam Pract.

[10] Anandkumar S, Manivasagam M, Kee VTS, Meyding-Lamade U (2018). Effect of physical therapy management of nonspecific low back pain with exercise addiction behaviors: a case series. Physiother Theory Pract.

[11] Cook B, Karr TM, Zunker C, Mitchell JE, Thompson R, Sherman R (2013). Primary and secondary exercise dependence in a community-based sample of road race runners. J Sport Exerc Psychol.

[12] Bratland-Sanda S, Sundgot-Borgen J (2015). “I'm concerned – what do I do?” recognition and management of disordered eating in fitness center settings. Int J Eat Disorder.

[13] Corazza O, Simonato P, Demetrovics Z, Mooney R, van de Ven K, Roman-Urrestarazu A (2019). The emergence of exercise addiction, body Dysmorphic disorder, and other image-related psychopathological correlates in fitness settings: a cross sectional study. PLoS One.

[14] Giordano S (2005). Risk and supervised exercise: the example of anorexia to illustrate a new ethical issue in the traditional debates of medical ethics. J Med Ethics.

[15] Rigotti NA, Nussbaum SR, Herzog DB, Neer RM (1984). Osteoporosis in women with anorexia nervosa. N Engl J Med.

[16] Marshman GM, Hanna MJD, Ben-Tovim DI, Walker MK (1990). Cutaneous abnormalities in anorexia nervosa. Australas J Dermatol.

[17] Lasater LM, Mehler PS (2001). Medical complications of bulimia nervosa. Eating Behav.

[18] Sim LA, McAlpine DE, Grothe KB, Himes SM, Cockerill RG, Clark MM (2010). Identification and treatment of eating disorders in the primary care setting. Mayo Clin Proc.

[19] Hoek HW, van Hoeken D (2003). Review of the prevalence and incidence of eating disorders. Int J Eat Disord.

[20] Alberga AS, Russell-Mayhew S, von Ranson KM, McLaren L (2016). Weight bias: a call to action. J Eat Disord.

[21] Cunningham HE, Pearman S, Brewerton TD (2016). Conceptualizing primary and secondary pathological exercise using available measures of excessive exercise. Int J Eat Disord.

[22] Lichtenstein MB, Emborg B, Hemmingsen SD, Hansen NB (2017). Is exercise addiction in fitness centers a socially accepted behavior?. Addict Behav Rep.

[23] Muller A, Loeber S, Sochtig J, Te Wildt B, De Zwaan M (2015). Risk for exercise dependence, eating disorder pathology, alcohol use disorder and addictive behaviors among clients of fitness centers. J Behav Addict.

[24] Lejoyeux M, Avril M, Richoux C, Embouazza H, Nivoli F (2008). Prevalence of exercise dependence and other behavioral addictions among clients of a Parisian fitness room. Compr Psychiatry.

[25] Stapleton P, McIntyre T, Bannatyne A (2016). Body image avoidance, body dissatisfaction, and eating pathology: is there a difference between male gym users and non-gym users?. Am J Mens Health.

[26] Levitt DH (2008). Participation in athletic Activitiesand eating disordered behavior. Eat Disorders.

[27] Prichard I, Tiggemann M (2005). Objectification in fitness centers: self-objectification, body dissatisfaction, and disordered eating in aerobic instructors and aerobic participants. Sex Roles.

[28] Wojtowicz AE, Alberga AS, Parsons CG, von Ranson KM (2015). Perspectives of Canadian fitness professionals on exercise and possible anorexia nervosa. J Eat Disorder.

[29] Manley RS, O'Brien KM, Samuels S (2008). Fitness instructors' recognition of eating disorders and attendant ethical/liability issues. Eat Disorder.

[30] Worsfold KA, Sheffield JK (2018). Eating disorder mental health literacy: what do psychologists, naturopaths, and fitness instructors know?. Eat Disorder.

[31] Billieux J, Schimmenti A, Khazaal Y, Maurage P, Heeren A (2015). Are we overpathologizing everyday life? A tenable blueprint for behavioral addiction research. J Behav Addict.

[32] Marks P, Harding M. ‘Fitness Australia Guidelines: Identifying and managing members with eating disorders and/or problems withexcessive exercise’. A collaborative project between The Centre for Eating & Dieting Disorders [CEDD] and Fitness First Australia on behalf of Fitness Australia. Sydney, Australia 2004.

[33] National Eating Disorders Association. Coach & athletic trainer toolkit. 2010. https://www.nationaleatingdisorders.org/learn/help/coaches-trainers. Accessed September 18 2019.

[34] ISRM Information Note No 352: Managing users with suspected health problems: eating disorders. 2010. https://emduk.org/wp-content/uploads/2017/01/Managing-Users-with-Suspected-Health-Problems-Eating-Disorders.pdf Accessed September 18 2019.

